# The Assessment and Response of Rehabilitation Professionals to Sudden Deterioration in Symptoms: An Analysis of the National Database in Japan

**DOI:** 10.1298/ptr.E10272

**Published:** 2024-02-10

**Authors:** Naoki SASANUMA, Keiko TAKAHASHI, Akiyo EGUCHI, Shinya YAMAUCHI, Yuki UCHIYAMA, Kazuhisa DOMEN

**Affiliations:** ^1^Department of Rehabilitation, Hyogo Medical University Hospital, Japan; ^2^School of Medicine, Department of the Patient Safety and Quality Management, Hyogo Medical University, Japan; ^3^School of Medicine, Department of Rehabilitation Medicine, Hyogo Medical University, Japan

**Keywords:** Rehabilitation, Patient safety, Resilience engineering, Functional resonance analysis method, Acute deterioration

## Abstract

Objective: There are few analyses of the current status of and responses to acute deteriorations encountered by physiotherapists, occupational therapists, and speech-language pathologists (rehabilitation professions [RPs]). The purpose of this study was to analyze the responses of RPs to acute deterioration in patients using the functional resonance analysis method (FRAM) based on the descriptions in “the Medical Accident Database”. Methods: Subjects were 413 cases with medical incidents reported by RPs to the database from 2012 to 2021. Life-threatening cases with changes in consciousness, circulation, and respiration were selected. Descriptions regarding findings assessed by RPs and support team, and requests for assistance were extracted. We also attempted to construct appropriate respond in RPs by using the FRAM. Results: Thirty-nine cases of acute deterioration were included in the analysis, and descriptions by RPs of consciousness (35 cases), circulation (18 cases), and respiration (36 cases) were identified. Blood pressure and percutaneous oxygen saturation measurement were frequently presented in the assessment by RPs, whereas the support team assessed cardiac arrest and respiratory arrest in high frequency. The FRAM analysis indicated that appropriate and rapid post-response by RPs requires patient information in prior, appropriate assessment and integration/interpretation. Conclusion: We attempted to identify problems analyzing the response by RPs to acute deterioration using the database and construct an appropriate response model. It resulted that RPs need to obtain patient information in advance and integrate/interpret it appropriately based on accurate assessment of conscious, circulation and respiration for rapid response. A model including integration/interpretation for appropriate post-response by RPs was constructed using the FRAM.

## Introduction

Cardiopulmonary resuscitation (CPR)^1–3)^ as a first response to a patient’s acute deterioration is an essential part of the medical community^4)^ and is being analyzed to further improve outcomes^5)^. One limitation of CPR is that the algorithm does not address the problem of adapting to the complex nature of resuscitation procedures^5)^. Shortcomings in rescuer performance have also been identified as a factor in poor CPR outcomes^5)^.

The incidence of adverse events involving rehabilitation professions (RPs) in the practice of rehabilitation medicine has been reported to range from 0% to 22%, with most of these being minor changes in vital signs^6)^. In a single-center retrospective analysis of patients’ deterioration, 9 adverse events (incidence rate of 0.032%) were reported over a 7-year period^7)^. Five of the adverse events in this report were life-threatening, including cardiopulmonary arrest, pulseless electrical activity, and loss of consciousness due to cerebral infarction and included a variety of lesions^7)^.

The Japan Council for Quality Health Care (JCQHC) shares information concerning medical safety measures as the National Database of Medical Adverse Events^8)^. This information includes both medical accident information and “near-miss” cases^9)^, and since the start of the project in 2004, information from various professions has been disclosed, including cases reported by RPs. Previously, many analyses have been reported on multidisciplinary cooperation, analysis of ventilator-related critical incidents, falls, and choking^10–14)^ in this database. In the database’s analysis of more than 57,000 incidents and accidents in which RPs were involved^15)^, trends were comprehensively analyzed, and especially, cases related to medical devices including mechanical ventilators and oxygen therapy equipment were carefully examined. This report described the need to share information and monitor the status of patients and equipment before, during, and after rehabilitation^15)^. On the other hand, this report descripted one of the limitations was that not enough refer to analysis of elements about rehabilitation situations occurring medical accidents^15)^. So far, there has been no report in the National Database of Medical Adverse Events of the JCQHC that fully examines the occurrence of life-threatening medical accidents to which RPs are a party and the problems in subsequent responses.

The functional resonance analysis method (FRAM) was developed by Hollnagel to analyze socio-technical systems and can be used in medical system analysis^16)^. The FRAM views a system from six aspects (input, output, preconditions, resources, control, and time), reveals problems when accidents occur, and has demonstrated its effectiveness in improving systems in the medical scene^17–21)^, including the rehabilitation field^22,23)^.

In this study, we used the FRAM to clarify the responses of RPs based on medical accidents reported to the National Database of Medical Adverse Events, as well as the current status and issues in medical accidents that RPs encounter related to life-threatening situations. The aim of the study was to clarify the assessment and subsequent response by RPs in the event of an acute deterioration, and to construct a model to lead to a more appropriate response.

## Methods

This study was approved by the Ethics Committee of Hyogo Medical University (control number 202301-003). Subjects were 413 medical accident reports involving RPs submitted to the National Database of Medical Adverse Events from 2012 to 2021.

The primary outcome was to clarify the assessment and subsequent response by RPs in the event of acute deterioration, and the secondary outcome was to construct a model to lead to a more appropriate response.

### Data selection

The National Database of Medical Adverse Events in the JCQHC includes the following data^8,15)^. Case ID, day of the week, day category, time of occurrence, whether or not medical care was provided, degree of treatment, degree of accident, place of occurrence, summary, cases requiring special reporting, related departments, number of patients, patient age, patient sex, patient category, name of disease directly related to the accident, patient’s condition immediately before the accident, person who found the accident, parties involved, professional categories involved, medical specialists/certified physicians and other medical personnel with specialties/certifications, experience in the professional categories involved (number of years), number of shifts and night shifts in the previous week, type of work, working hours in the previous week, scene of occurrence, related drugs_sales name, related drugs_manufacturer’s name, medical materials/supplies_sales name, medical materials/supplies_ manufacturer’s name, medical materials/supplies_date of purchase, medical equipment_ sales name, medical equipment_ manufacturer’s name, medical equipment_date of purchase, medical equipment_date of manufacture, medical equipment_date of most recent maintenance inspection, purpose of the medical treatment performed, case description, cause of occurrence (factors related to the behavior of the parties, human factors, environment/equipment, etc.), summary of case background factors, and whether or not an accident investigation committee has been established.

**Fig. 1.**
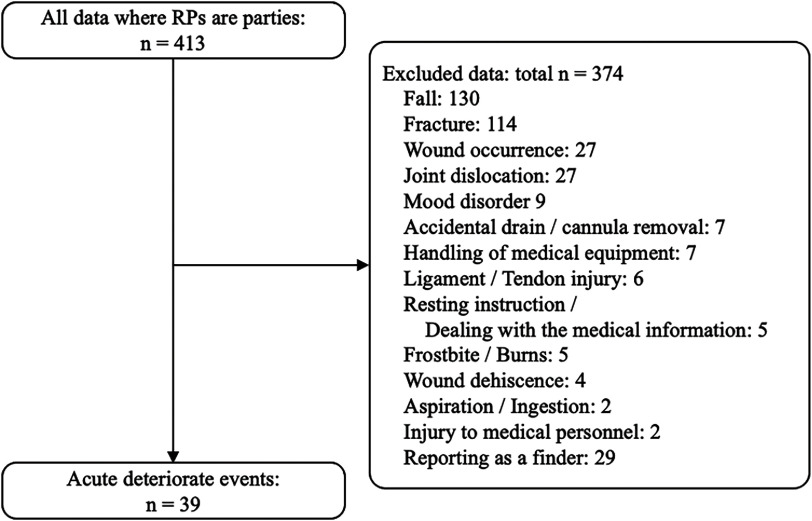
Extracted data flow RPs, rehabilitation professions

We extracted acute deterioration cases from a database of 413 cases by the following procedure. Step 1. The author, N.S., conducted a full-text screening of the database sections “Case Description” and “Summary of Case Background Factors” to exclude cases of falls, fractures, wound occurrence, and joint dislocations. Cases related to mood disorder, drains, catheters, medical equipment use, ligament/tendon injuries, resting instructions and medical information dealing, frostbite/burns, wound dehiscence, ingestion and aspiration of foreign objects, and injuries sustained by healthcare professionals themselves, and cases reported as the person who found the injury were then excluded. We extracted assessment descriptions in 39 cases with acute deterioration in consciousness, hemodynamics, and respiratory status. In addition, for the 39 cases, the full-text screening of the database sections “Case Description” and “Summary of Case Background Factors” was performed to extract the following information: descriptions of assessment items related to consciousness, circulatory status, and respiratory status; descriptions of responses taken by RPs after a sudden deteriorated event; descriptions of requests for support; descriptions of the responders; descriptions of assessment items conducted by responders and support teams; and descriptions of the support team’s response to the acute deteriorate event. Step 2. Secondary screening by co-authors S.Y. (physical therapist), Y.U. (rehabilitation doctor), and A.E. (cardiologist) was performed to validate the items indicated in Step 1. Step 3. Co-author K.T. (specialist in patient safety and medical quality management) verified the selected words, and finally, the target cases and each descriptive item were extracted by a four-person panel. The flow of data extraction is shown in Figure 1, and the extracted data are presented as Supplementary Table 1.

### Data analysis

Incident attributes (therapy type, therapists’ years of experience, year of occurrence, day of the week of occurrence, time of occurrence, degree of disability, age range of patients, related clinical departments) for the 39 cases were tabulated. The assessment items described by the RPs when they detected a sudden deterioration, and the assessment items described by the responders and support team were tabulated. Blood pressure and percutaneous oxygen saturation (SpO_2_) measurements were measured successfully or unsuccessfully, and the means by which the RPs requested support and the job title of the responder were tabulated to understand the actual status of the request made by the RPs.

### Functional resonance analysis

Functional resonance analysis was conducted to visualize the RPs’ actual responses based on the descriptions of the 39 cases and to construct a more appropriate response model. The “functions” were identified from the descriptions of what the RPs and the support team actually did (work as done)^24)^. In the FRAM, each function has six aspects: input, output, preconditions, resources, time, and control^16)^. These aspects were also presented based on the 39 case descriptions. Inputs are the items that caused the RPs or support team to perform the function. Outputs are the results of the functions actually performed by the RPs and the support team. Preconditions are the signals that, together with the inputs, cause the function to operate. Resources are the personnel, equipment, and information required for the function. Control describes the “decisions” and “rules/procedures” that affect the “output”.

The description of each function and aspect was conducted through the hierarchical cross-check method in accordance with the previous study^23)^. 1. The author N.S. extracted the actions taken by the RPs and the support team from the database. The author N.S. also extracted the items that were not actually performed but should have been performed, the risk factors the patient had, the decisions made by the RPs and support team, the resources used, and the rules and procedures followed/not followed from the “Summary of Case Background Factors” in the database. 2. Co-authors S.Y., Y.U., and A.E. checked the appropriateness of step 1, and if deemed inappropriate, step 1 was performed again. 3. Co-author K.T. finally checked the appropriateness of the “Functions” and elements were determined by consensus of the four members.

Following Sundstrom and Hollnagel’s procedure^25)^, each function was described and a functional resonance analysis model was constructed, which involved 4 steps: 1. identification of each function, 2. identification of each element, 3. identification of the connections (functional resonance) among the elements, and 4. graphical representation of the functional resonance analysis model. FRAM model visualizer^26)^ was used to construct the FRAM model.

### Statistical analysis

The number of patient assessment statements completed by the RPs and support team was counted for each category of consciousness, circulation, and respiration. A chi-square test was performed to compare the number of assessment counts between the RPs and the support team. Significance was set at <5%. SPSS ver. 12.0 J was used as the statistical analysis software.

## Results

### Characteristics of cases

A summary of the 39 cases from which the basic descriptive data were extracted is presented in Supplementary Table 1. The attributes of the 39 cases are shown in Table 1. Physical therapists were the most frequent type of therapist with 25 cases than occupational therapists and speech-language-hearing therapists. Nine cases were reported, by years of experience, in the age group category of 1 year or less, the largest number in the age group category, while the other categories ranged from 4 to 7 cases. Sixteen cases (41%) were reported in patients with less than 5 years of experience, while the same number of 16 cases were reported in patients with more than 10 years of experience. The number of cases by day of the week tended to be higher on Mondays, Wednesdays, and Fridays. The majority of cases were reported between 10:00 and 16:00.

**Table 1.** Characteristics of acute deteriorated cases shown in the National Database of Medical Adverse Events

Therapy type		Therapists’ experience years		Year of occurrence		Day of the week of occurrence		Time of occurrence		Degree of disability		Age range of patients (years)	
Physical therapist Occupational therapist Speech language hearing therapist	25 5 9	≤1 2–4 5–9 10–14 15–19 20≤	9 7 7 6 4 6	2012 2013 2014 2015 2016 2017 2018 2019 2020 2021	2 3 9 3 0 1 5 2 5 9	Sunday Monday Tuesday Wednesday Thursday Friday Saturday	1 9 3 13 4 8 1	6:00–7:59 8:00–9:59 10:00–11:59 12:00–13:59 14:00–15:59 16:00–17:59	1 2 13 9 13 1	No disability No possibility of residual disability Likelihood of residual disability (low) Likelihood of residual disability (high) Death Unknown	5 7 9 5 12 1	0–9 10–19 20–29 30–39 40–49 50–59 60–69 70–79 80–89 90≤	2 0 0 1 0 1 8 13 11 3
Related clinical departments (multiple election)													
Rehabilitation medicine	12	Respiratory Medicine	4	Psychiatry	2	Gastroenterology	2	Otorhinolaryngology	2	Gastroenterological surgery	1	Surgery	1
Internal medicine	4	Neurosurgery	3	Orthopedics	2	Pediatrics	2	Emergency medicine	2	Hematology	1	Rheumatology	1
Circulatory medicine	4	Respiratory surgery	3	Neurology	2	Cardiovascular surgery	2	Cardiovascular surgery	1	Plastic surgery	1	Unknown	1

Table 2 shows a comparison of the number of descriptions of the RPs’ and the support team’s evaluations. In the evaluation of consciousness, “no response” and “decreased level of consciousness” were frequently given, and relatively few were described in accordance with “open eyes,” “verbal response,” and “motor response,” as in the Japan Coma Scale and Glasgow Coma Scale. There were no differences between groups. In terms of the number of assessment of hemodynamics, blood pressure, heart rate, and pulse rate were most frequently described by RPs, in that order, and heart rate and cardiac arrest were most frequently described by the support team. Blood pressure was significantly higher in RPs, and cardiac arrest was significantly higher in the support team. Respiratory assessment was most frequently described by both the RPs and the support team for SpO_2_ assessment. Respiratory arrest was described in 8 cases by the support team (p = 0.038) compared to 3 cases by the RPs.

**Table 2.** Assessment items performed by RPs and support team on acute deteriorated cases

	RP findings n (%)	Support team findings n (%)	p value
Consciousness, total	35(89.7)	14 (35.9)	
Impression	24 (68.6)	6 (42.9)	0.116
EVM	11 (31.4)	8 (57.1)	0.116
Circulation, total	18 (46.2)	28 (71.8)	
BP measurement	12 (66.7)	5 (17.9)	0.001
Heart rate	3 (16.7)	9 (32.1)	0.315
Pulse rate	2 (11.1)	1 (3.6)	0.552
Pulse palpation	1 (5.6)	5 (17.9)	0.380
Cardiac arrest	0 (0.0)	8 (28.6)	0.015
Respiration, total	36 (92.3)	25 (64.1)	
SpO_2_ findings	15 (41.7)	9 (36.0)	0.791
Dyspnea	7 (19.4)	0 (0.0)	0.035
Respiratory rate Respiratory pattern	5 (13.9)	7 (48.0)	0.203
Respiratory arrest	3 (8.3)	8 (32.0)	0.038
Others	6 (16.7)	1 (4.0)	0.223

Numbers indicate the number of findings described.

RPs, rehabilitation professions; EVM, measurement according to eye opening, verbal response, and motor response; BP, blood pressure; SpO_2,_ percutaneous oxygen saturation

Table 3 shows the means of requesting assistance and the results for responders. Regarding the description of the means by which RPs request assistance, the largest number of unclear descriptions such as “call, contact, request, report” (15 cases) was followed by “no description” (8 cases). The most frequently mentioned means were “call button for nurse room” (8 cases) and “emergency calls” (4 cases) in the descending order of frequency. The most frequent responder was nurse (20 cases), and “no description” was stated in 9 cases.

**Table 3.** Means of requesting support and first responders

Means of requesting support	Numbers
Call button for nurse room	6
Emergency call	4
Loud voice request	1
Phone call	2
Doctors/Nurses in the vicinity	2
Nurse visits room with monitor alarm	1
Descripted only as “call, contact, request, report”	15
No description of means of requesting support	8
First responders	Numbers
Nurses	20
Doctors	5
Nurse and doctor	4
Therapists	1
No description	9

### FRAM model

**Fig. 2.**
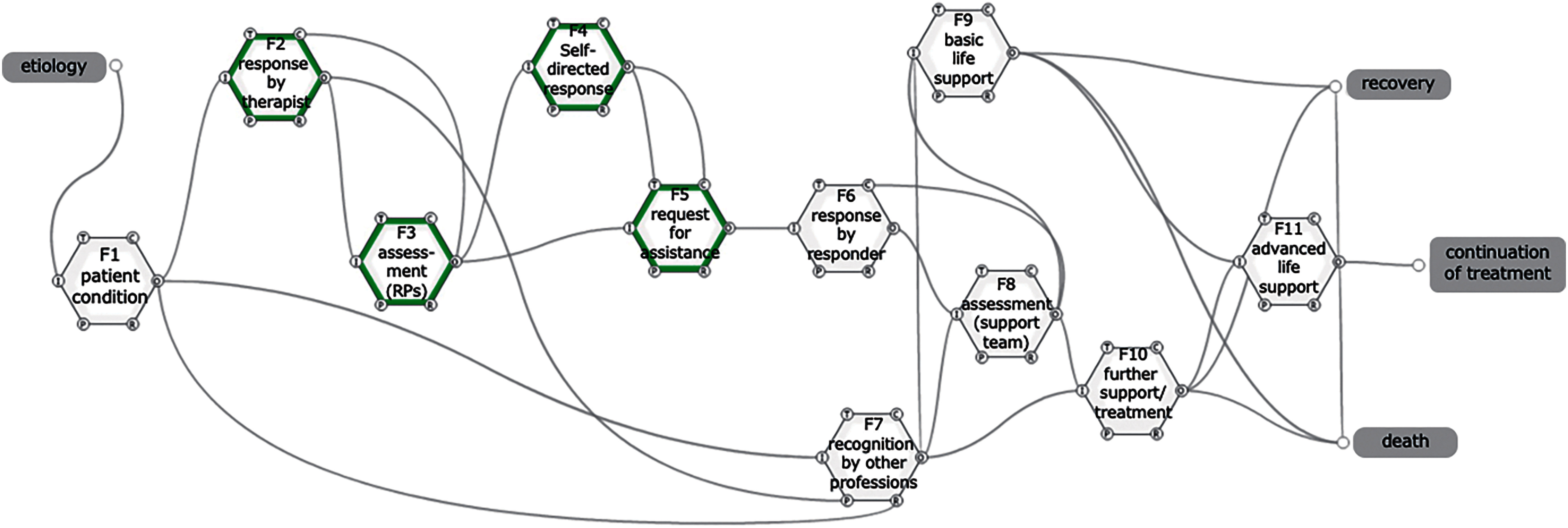
Functional analysis model based on actual descriptions F in green border indicates functions performed by RPs. RPs, rehabilitation professions; I, Input; O, Output; P, Preconditions; R, Resources; T, Time; C, Control

In this functional resonance analysis, the “function” was defined as what the RPs and the support team “actually performed” and was confirmed from the descriptions. The actual description and its relationship to each aspect or function is shown in Supplementary Table 1. In addition, the patient’s condition, which is the basic condition for rehabilitation by the RPs, was set up for model building. Eleven “functions” were set based on the “actually performed” taken by the RPs and the support team. These functions are listed in Table 4. The relationship between the functions is shown by linking the words of each element in Table 4. Figure 2 was created to facilitate understanding of the relationships among the functions.

**Table 4.** Each function of FRAM and six aspects of each function

	F1: patient condition	F2: response by therapist	F3: assessment (RPs)	F4: self-directed response	F5: request for assistance	F6: response by responder
Input	Pathological conditions	Patient status	First remarks	Assessment results	Assessment result	Request
Output	Patient status Noticing slight changes Monitoring equipment	First remarks	Assessment result No result obtained	Determination of need for backup	Request	Evaluation
Preconditions		Intended observation				
Resources						
Time				Time delay	Time delay	
Control		No result obtained				No result obtained
	F7: recognition by other professions	F8: assessment (support team)	F9: basic life support	F10: further support/treatment	F11: advanced life support	
Input	Noticing slight changes	Assessment	Severity	Severity	Treatment continued	
Output	Severity of illness Assessment	Severity No result obtained	Continue treatment Stable Irreversible lesion	Continuation of treatment Stable Irreversible lesion	Continuation of treatment Stable Irreversible lesion	
Preconditions	First remarks					
Resources	Monitoring equipment					
Time						
Control						

FRAM, functional resonance analysis method; F, function; RPs, rehabilitation professions

For example, “F3: assessment (RPs)” starts with “first remarks”, which is the “output” of “F2: response by therapist”, as “input”. “Assessment result” is output as a result of the assessment performed by the RPs in F3, it leads to the activation of “F4: self-directed response” or “F5: request for assistance.” On the other hand, if “F3: assessment (RPs)” outputs “no result obtained”, it is output to “control” of “F2: response by therapist” and becomes a control factor in the decision by the RPs to reevaluate or continue the therapy. The “no result obtained” of “output” in “F3: assessment (RPs)” is set based on the description that no result was obtained in 8 out of 12 cases in the blood pressure measurement results. Thus, a FRAM based on “actually performed” was created to model the behavior of RPs in clinical practice.

### Modified FRAM model

**Fig. 3.**
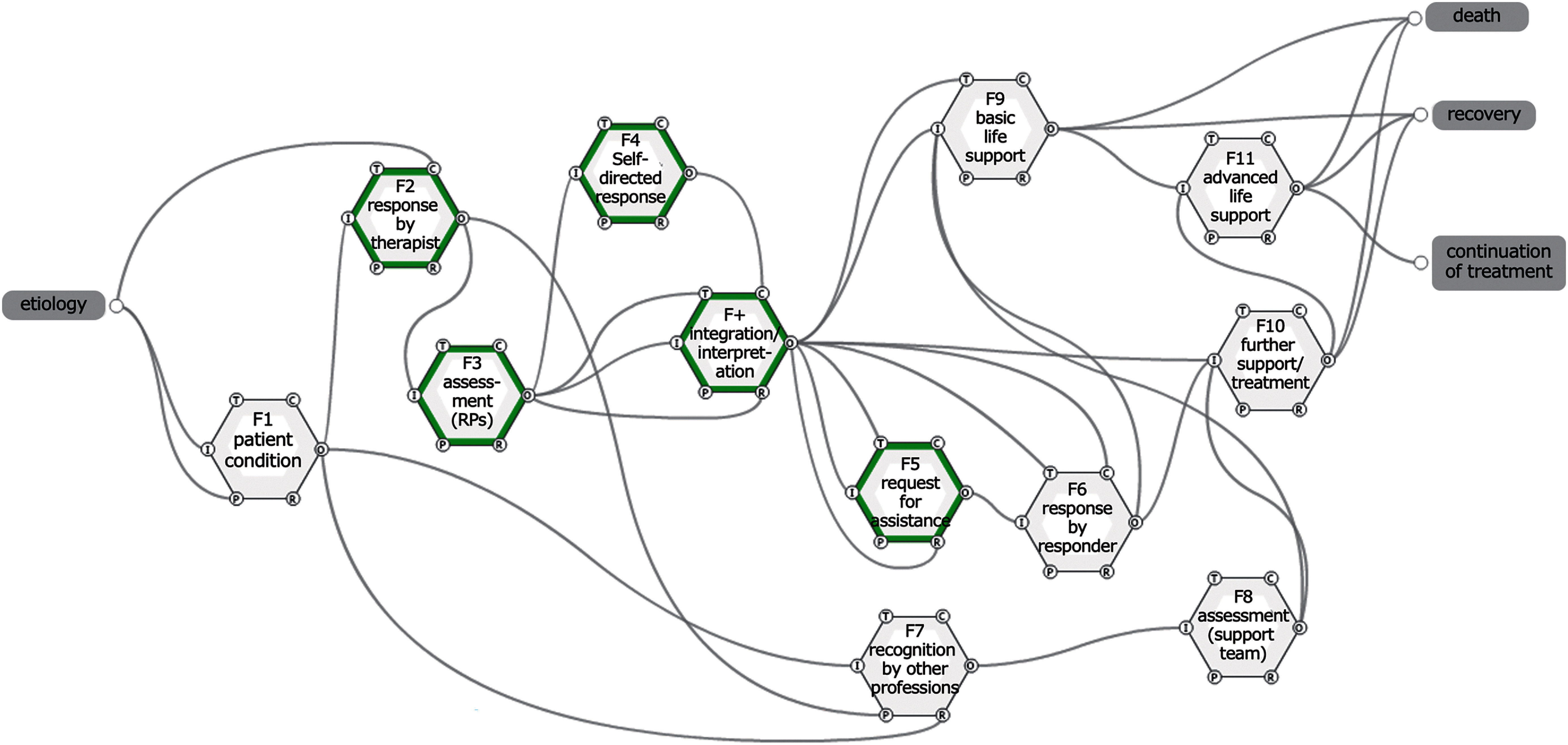
Modified functional analysis model F in green border indicates functions performed by RPs. RPs, rehabilitation professions

The "Summary of Case Background Factors" in the database also included descriptions of backward-looking analysis of incidents, such as "actions that should have been taken but were not actually taken" and "information that should have been known.” Therefore, to construct an appropriate response model using RPs, these descriptions were used to improve “functions” and each element, and a modified model was created. The functions and elements are listed in Table 5, and the relationships among the “functions” are shown in Figure 3. The patients’ background and pathological conditions are listed in Table 6, and elements in Table 6 correspond to “preconditions” of F1 in Figure 3.

**Table 5.** Modified FRAM and six aspects of each function

	F1: patient condition	F2: response by therapist	F3: assessment (RPs)	F4: self-directed response	F5: request for assistance	F6: response by responder
Input	Pathological conditions	Patient status	First remarks	Modulation other than consciousness, respiratory, and circulation	Necessity of request	Request
Output	Noticing slight changes Patient condition Monitoring signal	First remarks	Assessment result Appropriateness of assessment Judgment of urgency	Judgment of need for backup	Request	Severity
Preconditions	Influencing factors (see Table 6)	Confirmation with physician, decreased risk perception			Initiating response upon discovery	
Resources	Monitoring equipment	Information about the patient, findings from observations, personnel			Appropriate means	
Time		Frequent observation			Urgency	Urgency
Control	Rules/procedures (use of elastic stockings)	Family factor (strong desire for feeding) Rules/procedures (suction-related, exercise load-related, confirmation of physician’s orders) Decision (decision not to observe, need for suctioning)	Frequent measurements	Judgment that suctioning would improve the situation	Unaware of emergency nurse call	Appropriate communication of assessment results
	F7: recognition by other professions	F8: assessment (support team)	F9: basic life support	F10: further support/treatment	F11: advanced life support	F+: integration/interpretation
Input	Patient condition	Assessment	Severity	Severity	Treatment continued	Assessment results
Output	Assessment	Severity	Continue treatment Stable Irreversible lesion	Continuation of treatment Stable Irreversible lesion	Continuation of treatment Stable Irreversible lesion	Necessity of request, Appropriate communication of evaluation results, Urgency, Severity, Appropriate means, Modulation other than consciousness, Respiratory, Circulation
Preconditions	First remarks					
Resources	Monitoring equipment			Information (neurologist) Information (outpatient nurse)		Appropriateness of assessment
Time			Urgency			Judgment of urgency
Control						Judgment of need for backup

FRAM, functional resonance analysis method; F, function; RPs, rehabilitation professions

**Table 6.** Details of “influencing factors” in F1, preconditions

General condition	Coagulation system
Advanced age Poor general condition Severely ill Not extremely low in food or water intake Circulation History of heart disease Decreased cardiopulmonary function Rupture of intercostal artery aneurysm Hemorrhagic shock Abnormally thin anterior wall Respiration Decreased respiratory function Airway obstruction Risk of asphyxiation Frequent pneumonia (aspiration pneumonia) Large amount of viscous sputum High expectoration of sputum Viscous phlegm Large amount of sputum Need to aspirate SpO_2_ drops during suction Malignant tumors Tumors Lung cancer Under radiation therapy Patients with cancer	D-dimer Pathophysiology predisposing to thrombosis DVT present Cognitive function Dementia Unclear responses Eyes open Poor response Swallowing function Ingestion Aspiration pneumonia Aspiration risk Decreased swallowing ability Activity Decreased ADL Lack of strength in lower limbs Motor paralysis Difficulty in physical movement Sensation Pain in the lower extremities Numbness in the lower extremities Sensory disturbance Other Desires of the person

F, function; DVT, deep vein thrombosis; ADL, activities of daily living; SpO_2_, percutaneous oxygen saturation

The following is an example of a specific input. In “control” of “F2: response by therapist”, “family factor” and “rules/procedures” are listed. Cases in which strong family requests are one of the controlling factors for RPs’ behavior (case 6 in Supplementary Table 1), adherence to rules established by the organization or team (e.g., case 11 in Supplementary Table 1) and deviation from procedures (e.g., case 12 in Supplementary Table 1) are the basis. Table 5 and Figure 2 show that the output “first remarks” of “F2: response by therapist” is input to “F3: assessment (RPs).”

In the modified model, “F+: integration/interpretation” was set as the destination function of the output from “F3: assessment (RPs).” In Table 4 and Figure 2, the two outputs from “F3: Assessment (RP)” are “assessment result” and “no result obtained.” On the other hand, in the modified model, the outputs were “assessment result,” “appropriateness of assessment,” and “judgment of urgency,” which were output as time factors and preconditions for the subsequent “F+: Integration/interpretation.” “F+: Integration/interpretation” was constructed as a process in which the “assessment result” from F2 was used as input, and “necessity of request,” “appropriate communication of evaluation result,” “urgency,” “severity,” “appropriate means,” and “modulation other than consciousness, respiratory, circulation” were outputs.

## Discussion

In this study, we investigated medical accidents involving RPs disclosed by the National Database of Medical Adverse Events of the JCQHC to analyze cases of sudden deterioration. The results confirmed that about half of the RPs encountering acute deteriorated cases were in their 5th year or younger and that most of them occurred during the daytime on weekdays with severe outcome.

The RPs frequently described blood pressure measurements, and the support team frequently described cardiac arrest and respiratory arrest. The description of the means of requesting support by RPs was limited, and the majority of responders were nurses, confirming the reality of clinical collaboration.

Functional resonance analysis confirmed that the evaluation in the RPs influenced the next call for support and other actions, suggesting the need for a function that integrates and interprets the appropriateness of the evaluation and multiple results as a modified model.

In this database analysis, differences in the number of cases by year groups of experiences were limited. In a previous study^15)^, in an analysis of near-miss cases involving rehabilitation professionals and cases related to oxygen therapy equipment, it was concluded that most of the parties involved were within 3 years of graduation. On the other hand, Kinoshita et al.^7)^ reported that the number of years of experience of the parties involved was not related to the sudden change in patients. Our study showed that many patient factors, compliance with rules, appropriate assessment, and prompt response are necessary to respond to the acute deterioration. It was suggested that those with fewer years of experience may not be able to adequately assess the situation based on an understanding of these multiple factors when dealing with patients. The relationship between years of experience and medical accidents encountered may differ depending on the type and nature of the accident, and we believe that continued investigation is needed.

The descriptions indicated that the acutely deteriorated cases encountered by the RPs had a mortality outcome of 12 cases or more. This result suggests the importance of the initial response made by the RPs. Many RPs learn Basic Life Support (BLS) as a basis for responding to emergencies in their pre- and postgraduate education^27)^. However, the present analysis confirmed that there were situations in which the BLS algorithm was not the first choice to be initiated in the event of an emergency at a medical institution (for example, in cases 2, 9, 10, 11, 13, 14, 18, 21, 24, 25, and 26 in Supplementary Table 1). In these cases, the parties involved were required to select various actions, such as patient observation and call for support, as well as the implementation of possible recovery measures. Limitations of the BLS algorithm have been noted in that it does not address complex situations where the need for resuscitative measures arises^5)^. To select the appropriate action, the RPs may need to be able to observe slight changes in signs; quickly and appropriately evaluate findings of consciousness, circulation, and respiration; determine whether to take recovery measures or request assistance; and if necessary, take immediate primary life-saving measures.

In the assessment of consciousness, the most common descriptions by RPs were “decreased level of awareness” and “response/non-response” (24 cases). The Japan Coma Scale and Glasgow Coma Scale, widely used consciousness assessment scales, include eye-opening state, verbal response, and motor response as measures of consciousness. In the present results, there were 11 cases in which one of these items was included in the description of consciousness, and there were no cases in which all three items were included. Observations based on these measures may be necessary to capture slight changes in consciousness. Blood pressure measurement was used for circulatory assessment in 12 cases, but measurement results were obtained in only 4 cases, and pulse measurement was described in only 3 cases (cases 2, 12, and 36). Of these cases, the description of pulse measurement by palpation was confirmed in one case (36). In order to obtain appropriate evaluation results, pulse palpation prior to blood pressure measurement is necessary^28)^. The present results suggest that palpatory pulse evaluation preceding blood pressure measurement may not have been sufficiently performed in the initial evaluation in physical therapists (PT), occupational therapists (OT) and speech-language-hearing therapists (ST). SpO_2_ measurements were frequently used in both PRs and support teams for respiratory assessment; support teams had about the same number of evaluations of respiratory rate and respiratory pattern, while RPs had fewer evaluations. SpO_2_ has been shown to miss the majority of apnea alerts^29)^. Recognition of cardiopulmonary arrest is based on the state of consciousness and respiratory effort^30)^, and the agonal/abnormal respiration pattern is important for recognizing cardiopulmonary arrest^2)^. Guidelines for the evaluation of spontaneous breathing^31)^ indicate the need for rapid and appropriate evaluation of thoracic movements. Assessment of breathing patterns in addition to SpO_2_ may be necessary for accurate assessment of respiratory status.

The use of emergency call as a means of requesting assistance was described in 4 of the 39 cases. A modified early warning system (MEWS)^32)^ has been presented as a criterion for using emergency call, and MEWS includes heart rate, respiratory rate, central nervous system findings, body temperature, urine output, and blood pressure^33)^. The clinical judgment of the healthcare professional is considered important for the appropriate use of MEWS^34)^. Therefore, RPs may require abilities to perform to these diverse assessments quickly.

In the functional resonance analysis, an initial model was constructed based on the “actual actions” identified from the descriptions, and a modified model was constructed by adding “things that should have been considered” and “possible factors” identified from the descriptions in the database. In the previous report^35)^, the necessity of understanding the factors causing the gap between “work-as-done” and “work-as-imagined” in the system and taking measures to eliminate the gap were discussed.

In this study, many “patient factors” and “factors contributing to clinical judgment” were identified in the modified model. The possibility that the RPs could encompass these elements prior to the assessment of patient response and acute deterioration, thereby allowing for proper implementation of the assessment and integration and interpretation of the assessment results, as part of clinical reasoning, was demonstrated. While evaluating, RPs are self-monitoring not only the presence or absence of results but also the appropriateness of the evaluation. As a result of this modification, it was shown that it is possible to move to “function,” such as promptly requesting support, giving information to the responder, and initiating BLS by oneself. It has been reported that fluctuations in two or more respiratory and circulatory indices are more useful in predicting the deterioration of a patient’s condition than a single index^36)^, and it is reasonable to integrate multiple findings into a single index^37)^. In addition, previous studies have reported that deriving appropriate interpretations from the information collected by healthcare providers can lead to favorable patient outcomes^38)^ and prevent worsening of disadvantage in the event of an event^39)^.

In Figures 2 and 3, “F7: recognition by other professions” was set. The presence of collaborators was shown to speed up the recognition of subtle changes and the transition to the next action. The effectiveness of multidisciplinary collaboration and monitoring devices was also confirmed in this model. In particular, nurses were the most common responder to RPs (20 cases), confirming the reality of collaboration in their work.

The importance of multidisciplinary collaboration has been reported in resuscitation in life crises^40–42)^ and in rehabilitation^43–47)^. The results suggest the need for RPs to acquire the ability to respond to unexpected events, and to practice responding promptly and appropriately together with other professionals.

There are several limitations to this study. We attempted to analyze cases listed in the National Database of Medical Adverse Events and considered about evaluation and response, depending on the descriptions in the report. In 2023, there are 180,732 medical facilities in Japan^48)^, but only 1158 (about 0.6%) of them have reported^49)^ to the database. Therefore, the disclosed cases are only a part of the outbreak cases, and there may be bias among medical institutions in their contents. Although we tried to reveal the earliest moment response of RPs who encountered patients in acute deterioration in symptom, this analysis based on the medical accident database may not reflect the real clinical situation or appropriate evaluations and responses were in fact made even if the information was not listed in the database. The measurement of individual and team interactions in actual emergency situations, which are not disclosed as descriptive items, is a future task. We included only 39 cases in the analysis. On the other hand, the 379 cases that were excluded, especially the cases of falls, fractures following falls, and brain injuries, which were the most frequently reported cases, may lead to serious consequences, and detailed analysis is required for these cases as well. In the future, more cases should be included in the analysis.

There are also limitations in the data sampling and FRAM model building process. The process of converting the database descriptions into data and the model construction based on the data were hierarchically cross-checked by multiple authors based on previous studies^23)^ in order to ensure objectivity.

The RPs and support team findings in three categories such as consciousness, circulation, and respiration were statistically analyzed. Because of the small number of cases in each group, results of statistical significance must be cautiously generalized.

The definition of function in the FRAM has been reported, including a multidisciplinary focus group study from multiple facilities^22)^, construction based on information from observation of work procedure and semi-structured interviews with healthcare professionals^18)^, or setting by coding of collected cases^20)^. No objective evaluation of the superiority or validity of these definitional procedures has been conducted in our knowledge. It may be necessary to examine the validity of the hierarchical cross-checking method in this study. The validity and appropriateness of this model have not been verified for the modified model constructed based on the FRAM analysis. The validation of the modified model to improve the ability of RPs to respond to sudden deterioration in patients is an issue for the future.

The modified model incorporates the background factors of each event in the database and is useful for understanding the actual situation of a patient deterioration in a real clinical setting. The RPs can be used in this model to prepare for the patient care in a clinical setting, to anticipate possible deterioration, to assess what to do in the acute event, and to anticipate the specific procedures for calling for assistance. In addition, this model can be applied to the creation of a simulation training program for sudden deterioration in a patient. We believe that this model will contribute to improving the performance of RPs when they encounter life-threatening events that are difficult to encounter in a daily clinical situation.

In this study, based on reports of medical accidents involving RPs, we examined the National Healthcare Adverse Events Database of the Japan Agency for Health Care Excellence and verified the actual response of RPs in the event of a sudden deterioration in a patient. The results of this analysis suggest the need for RPs to prepare for sudden patient changes by obtaining patient information in advance; integrating and interpreting it appropriately based on accurate assessment skills of conscious, circulation, and respiration; and quickly transitioning to the best post-response measures, including emergency BLS if necessary.

## Conflict of Interest

The authors have declared that no conflict of interest exists.

## Supplementary Material

Supplementary Table 1. The descripted database.
