# Supplementary material for: The Assessment and Response of Rehabilitation Professionals to Sudden Deterioration in Symptoms: An Analysis of the National Database in Japan
Source: Phys Ther Res. 2024 Feb 10;27(1):21–34. doi: 10.1298/ptr.E10272 (PMC11057387; doi:10.1298/ptr.E10272)
Supplement: Supplementary Table 1. — The descripted database. [file ptr-27-21-s01.pdf]

Table 1. The descripted database

Case #	Descripted findings recorded by first responder				Immediate actions of first responder	Means of requesting support	Responded supporters	Patient findings recorded by medical team				Post-support lifesaving response	Background factors: regarding the patients themselves	Background factors: non-patient	Classification of background factors: non-patient
	Consciousness	Circulation	Respiration	Other findings				Consciousness	Circulation	Respiration	others				
1	Unconsciousness	Blood pressure unmeasured	Respiratory arrest, SpO_2 measurement not available	Cyanosis	Request for assistance	Called therapist., Reported to rehabilitation doctor, Called the ward	rehabilitation doctor				(undescribed)	Bag valve mask ventilation, Oxygen administration, Cardiac massage., Transfer to treatment room		Time lag between arrival from the ward and therapist response<br>Therapy room is large, with oxygen tanks and stretchers (in the training room exit door, about 10+ meters from the relevant patient location, resuscitation kit, first aid cart, etc. not located on the floor)<br>Neurology physician was unaware of this.<br>Outpatient charge nurses were not familiar with the placement of supplies	Human factors (observation, judgment):C. R<br>Facility factors: R<br>Equipment Factors: R<br>Information (neurologist): C<br>Information (outpatient nurse): C
2	No loss of consciousness	Blood pressure measured, Pulse measured	No dyspnea	Vomiting	Observe condition, Request for support	Called to pick someone up	nurse			wheezing		Proceed to radiology.		The physiotherapist in charge treated other patients<br>Suctioning was not performed during rehabilitation.	Human factors (observation and judgment): C, R<br>Rules/procedures: C
3			Abnormal respiration		Seek means of support, Request for support	Called someone	nurse				Check vitals	Further call, Bag valve mask ventilation by physician, Intubation, Ventilate.		Need to review suctioning procedures.<br>I was away from the patient."	Rules/procedures: C<br>Judgment: C<br>Observation: C, R

4	Cessation of response,<br>Cessation of body movement				Request for support	Called	nurse			Suctioning.,<br>Cardiopulmonary arrest	Older age: C<br>Decline in ADL: C<br>Dementia: C<br>Unclear responses: C<br>Frequent pneumonia (aspiration pneumonia): C<br>History of heart disease: C		
5			Sputum retention,<br>Decreased oxygen saturation,<br>Weak respiration		Call for backup	request	Respondent's job title not stated	Cardiac arrest	Respiratory arrest	CPR.,<br>Intubation			
6				Vomiting	Vomit was expelled together with the doctor.,<br>Further suctioning.	someone in the room with you	Attending Physician		Respiratory status poor	Resuscitation.	Prone to choking: C<br>Aspiration pneumonia: C<br>Aspiration risk: C<br>Risk of choking: C<br>The person's wishes: C	Changed to regular diet at family's request. Family member's request."	Information (information about ability to chew and swallow): R<br>Information (sufficient amount of information for family to understand): R<br>Judgment: C<br>Understanding of the family's level of understanding: C
7	No response	HR in the 80s	SpO_2 decreased		Backup required	nurse call	nurse	HR in the 60s	Unable to measure SpO_2	Call for emergency medical attention., CPR	Decreased respiratory function: C<br>Decreased cardiopulmonary function: C<br>SpO_2 drops during aspiration: C<br>Viscous sputum volume: C<br>Need for suctioning (decreased coughing ability): C	ST alone was performing suctioning and checking patient condition<br>Needed to suction (decreased coughing ability)	Human factors: C, R<br>Judgment: C
8			Oxygen saturation down to 60s		Jackson Reese administered oxygen (responder undescribed)	(undescribed)	(undescribed)		O_2 saturation restored.,<br>Respiratory status is restored.	ICU admission.	Tumor: C<br>Airway obstruction: C		

9	Decreased consciousness	Blood pressure measured	SpO_2 measured	Pale face	Transferred to ward room by stretcher (responders undescribed)	(undescribed)	(undescribed)	Recovery of consciousness, Loss of consciousness again	Cardiac arrest	Respiratory arrest		Start resuscitation.	D-dimer 12.0: C<br>Pain in lower extremities: C<br>Numbness in lower extremities: C<br>Lower extremity weakness: C		
10	Decreased level of consciousness				The patient was placed in a supine position, Request for backup	call	nurse, medical doctor		Blood pressure not measured., Pulse is weak.	Temporarily responsive to breath sounds, SpO_2 not measured.		The patient loses consciousness again., Pulse is not palpable., Cardiopulmonary resuscitation., Code blue requested.	Ruptured intercostal artery aneurysm: C<br>Hemorrhagic shock: C		
11	Loss of consciousness				Confirm vitals, secure route, and make an emergency call to the all section of the hospital (responders undescribed)	(undescribed)	(undescribed)				Check vitals	Root secured., Emergency call to hospital.		Stockings were used to prevent the patient's coughing.<br>D-dimer not measured before postoperative rehabilitation.	Rules/procedures: C<br>Information: C, R
12	Loss of consciousness	Pulse measured	Effort-like breathing, Shortness of breath		Request for backup	In-hospital emergency call, Calling Out to Surrounding Areas	therapist		Pulse not palpable.			Move patients to bed., BLS	Abnormally thin anterior wall: C	No confirmation of availability of medical attendance was made by the attending physician, and the patient walked alone at the discretion of the physiotherapist in charge of the patient.<br>Started on bicycle ergometer (0.2-0.3 kg load)	Rules/Procedures: C<br>Judgment: C<br>Information: C
13			Respiratory distress, SpO_2 decreased		(undescribed)	(undescribed)	rehabilitation nurse					Contact ward nurses and attending physician., Cyanosis, Loss of consciousness (eye rolling), Use bag valve			

												mask, Suctioning			
14			Respiratory arrest, SpO_2 dropping		Request for backup	nurse call	medical doctor, nurse					Bag valve mask, Phlegm aspiration, Ventilator reconnected, SpO_2 restored.		Did not know there was an emergency nurse call	Information: C nurse call: R
15				Hangman found	Contact nurse	phone call	medical doctor	Pupils dilated	No heartbeat, No blood pressure readings			Attempt resuscitation with cardiac massage		Time of day when eyes were away from the occupational therapist	Human Factors: R Judgment: C Observation
16	Decreased consciousness, Upper extremity spasms, Left co-morbid bias		Shortness of breath, Respiratory arrest	Vital measurements, Facial pallor	Request for backup, Start preparation of first-aid cart and AED	Emergency call	medical doctor, nurse				(undescribed)	CPR	The patient did not have extremely low food or fluid intake: C	Early detection of patient condition change Immediate ward contact, emergency call, and preparation of necessary supplies such as emergency cart, etc. implemented No problem in handling the situation.	Observation: P Rules/Procedures: C Equipment Factors: R Judgment: C
17				CPA	undescribed	(undescribed)	(undescribed)				(undescribed)	Emergency resuscitation	Lung cancer: C Undergoing radiation therapy: C	No check on the patient's physical condition	Observation: C Judgment: C
18	Decreased consciousness	Blood pressure unresponsive			Elevate lower limbs and begin oxygen inhalation	Code blue requested	(undescribed)				(undescribed)	CT scan of head	Eyes open: C She's not responding well: C	Judged to be unable to continue rehabilitation	Judgment: C
19	Decreased consciousness		Cough, SpO_2 dropping		Request for backup	Requested assistance	nurse	Eyeballs supinated, No call response	Cardiac arrest	Shallow respiration, Respiratory arrest		Chest compressions, Code blue requested., Transfer to ICU.	Decreased ability to swallow: C	Insufficient observation	Observation: C
20	No response, Ocular supination, Weakness		Dyspnea	Dizziness	Transfer to stretcher and begin life-saving treatment (responder undescribed)	undescribed	(undescribed)				(undescribed)	Transfer to stretcher., Patient receiving life support., Entering CCU.		Risk perception decreased due to discharge the next day. Performed because of need to ascend and descend stairs on return home Nurse and physical therapist	Judgment: C Rule/Procedure: C

														changed rehab content without doctor's order	
21	Decreased consciousness	Unable to measure blood pressure		Headache, Sweating	Request for backup	Nurse call	nurse				(undescribed)	(undescribed)			
22	Eyeballs elevated, Decreased consciousness	Blood pressure low			The patient was returned to bed with a nurse	(undescribed)	nurse	Decreased level of consciousness	Cardiac arrest	Decreased SpO_2		Resuscitation.			
23	Loss of response., Weakness				Tried to check patient's state of consciousness., A nurse came to the room when the patient's consciousness was checked.	Checked the monitor and visited the room voluntarily	nurse	Decreased level of consciousness	HR 30 on ECG, Carotid pulse not palpable	No respiration		Requested loudly, Other nurses visit, Cardiac massage., Code Blue issued.			
24	Motor arrest, Decreased consciousness	Electrocardiogram confirms heartbeat, Blood pressure unresponsive	Breath sounds not audible		Request for backup	Request for backup	nurse		Waveform cardiac arrest			Oxygen administered, Administer fluids, Suction		Wearing biometric monitor. Observe the patient's condition Frequently measured vital signs for blood pressure fluctuations	Observation: R<br>Observation: C<br>Observation: C, T<br>Judgment: C
25			Tachypnea, SpO_2 low	Sweating	Put the patient to bed immediately., Request for backup	Nurse call	nurse			No improvement in oxygenation		Report to physician.			
26			Excessive sputum, SpO_2 decreased		Request for sputum suction	request	nurse			SpO_2 measurement not available		Commence suctioning., Respiratory status not improving., Begin CPR., Begin ventilatory management.	Excessive phlegm: C	Rules not followed. Patient has been self-aspirating since suctioning prior to intervention Not following the rules and leaving meals unattended Cases of aspiration and choking immediately	Rule/Procedure: C<br>Observation: C<br>Judgment: C<br>Information: R

										after eating occurred in the same ward at the same time, but the risk was not predicted.	
27	Decreased level of consciousness	Deterioration of respiratory status	Request for backup	contact	nurse		Cardiac arrest	Respiratory arrest	Begin chest compressions., Suctioning., Resume cardiac rhythm., Intubate., Respiratory management.	We were aware that the patient's respiratory condition was gradually worsening day by day. Information was not shared face to face with the nurse in charge on the day of the incident. Endotracheal suctioning was not performed due to the invasive nature of the procedure. Did not ask the rehabilitation physician to decide whether or not to conduct the training. Oxygen 5litteles was administered by mask, but care was given while floating the mask	Information: R<br>Information: C<br>Judgment: C<br>Judgment: C<br>Rules and Procedures: C<br>Response: C
28			Request for backup	Nurse call	nurse	Poor communicatio n, JCS 3 digits		Respiratory arrest	Airway secured., No resumption of breathing, Heartbeat., Carotid artery not palpable., Start sternal compressions, Bag-valve mask ventilation.,		

												Speech detected.			
29	Loss of consciousness			Blurred vision, Convulsions, Vomiting, Incontinence	Request for backup	(undescribed)	nurse					Transferring to stretcher., Transferring to treatment room.	Advanced age: C		
30		Decreased arterial pressure	Decreased SpO_2		(undescribed)	(undescribed)	medical doctor					Patient is receiving fluids., Bag-valve mask ventilating.	Poor general condition to begin with: C		
31	Eyes open, Unresponsive to vocalization, Decreased level of consciousness				Request for backup	called	nurse		Unable to measure blood pressure, ECG attached, waveform not confirmed.	Movement of the thorax is detected., No movement of thorax.		Doctor on call, Treatment, Infusion			
32		Unable to measure blood pressure	Decreased oxygenation		Request for backup	contact	medical doctor, nurse					Oxygen therapy	High viscosity of phlegm: C<br>High volume of sputum: C	Frequent suctioning required	Response: C, T
33	Weakness, Unconsciousness				Request for backup	request for cooperation	nurse		Blood pressure, Pulse rate	Respiratory rate.		Call physician, PEA, Chest compressions, ICU admission	Motor paralysis: C<br>Sensory disturbance: C	Several hours after bed rest was lifted	Information: R<br>Judgment: C
34			Lung murmur., Decreased SpO_2		reported to nurse	report	nurse			Low SpO_2		Report to physician, Reintubate			
35	Loss of consciousness		Respiratory distress		Request for backup	asked for help	nurse, medical doctor					Administer oxygen, Call for further help, Patient regains consciousness.	DVT present: C	DVT is known to be present	Information: R
36	Decreased consciousness	No radial artery palpable, Blood pressure unmeasurable		Urinary incontinence, Pale face	Request for backup	emergency call	(undescribed)					Transfer to HCU.	Difficulty moving: C	Deep vein thrombosis/pulmonary thromboembolism prophylaxis evaluation was ambiguous (adherence to rules was not uniform in the hospital)	Rules and procedures: C<br>Information: R<br>Judgment: C

													Venous echocardiography denied venous thrombosis in both lower extremities.
37			Respiratory distress, Decreased SPO2		Returned to the room in a wheelchair., Request for backup	Nurse call	nurse			Low SpO_2	Start 10L oxygen with mask, Transfer to treatment room.	Cancer patients: C Conditions that predispose to thrombosis: C	
38	Decreased consciousness, Decreased level of consciousness	Blood pressure, Heart rate	SpO_2 measurement not available	Peripheral cold sensation	Cannula flow increased., Called for backup as consciousness level did not improve after observation (maximum 7 minute).	called	emergency physician, rehabilitation doctor		Blood pressure measurement, Heart rate measurement		Transfer to stretcher, Return to ward.		
39		Blood pressure measurement	High sputum volume		A nurse is present and becomes aware of bradycardia and asystole, Wheelchair reclined.	accompanying	nurse		Bradycardia, Waveform flat		Call for further backup, CPR, Restart heartbeat.	Critical illness with poor general condition: C	
Corresponding "Function"	F3: assessment (RRs)				F4: self-directed response F5: request for assistance	F5: request for assistance F7: recognition by other professions	F6: response by supporters	F8: assessment (support team)			F9: basic life support F10: further support / treatment F11: advanced life support	Original data for item Preconditions, Resources, Control, Time and F+ in Table 5	

ADL: Activities of Daily Living, BLS: Basic Life Support, CCU: Coronary Care Unit, CPA: Cardiopulmonary Arrest, CPR: Cardiopulmonary resuscitation, CT: Computed Tomography, DVT: Deep Vein Thrombosis, ECG: Electrocardiogram, F: Function(s), HCU: High Care Unit, HR: Heart Rate, ICU: Intensive Care Unit, JCS: Japan Coma Scale, RPs: Rehabilitation Professions, SpO_2: percutaneous oxygen saturation
